# Lemon Juice in Acute Rheumatism

**Published:** 1852-01

**Authors:** 


*Lemon Juice in Acute Rheumatism.*—Dr. Babington, of Guy's Hospital, London, reports in the London Lancet, 8th November, 1851, several cases of acute rheumatism, treated with lemon juice, in which "he found it answer the most sanguine expectations." "The lemon juice which Dr. Babington employed was procured from a wholesale confectioner, the price being no more than sixpence per pint, when retailed in small quantity, and considerably less when furnished by the gallon. When Dr. Babington commenced making trials of the lemon juice, he prescribed it according to the recommendation of Dr. Owen Rees, in doses of from one to two ounces, three times a day, for an adult. More recently, however, he had ordered not less than three ounces, and much more—usually six ounces—taken at a draught, and without any admixture, three times a day. It might be supposed that the patient would find some difficulty in drinking so large a quantity of such a sour liquid; but this is rarely the case, nor does it in general produce tormina, nor otherwise disagree with the alimentary canal. Instead of relaxing the bowels, as might have been expected, it renders them somewhat costive, so that it not unfrequently becomes necessary to exhibit an aperient. The juice produces no very decided effect upon the kidneys, merely tending, by its quantity, to promote their secretion. It increases cutaneous action, but to what extent it is difficult to determine, because, in rheumatism, a disease in which this remedy is chiefly useful, the complaint itself is marked by the occurrence of profuse perspiration. The only unequivocal effect which uniformly takes place is a diminution in the number and power of the pulse, and of the heart's action. Whether it alters the character of the blood, or whether it affects the heart by diminishing nervous influence, Dr. Babington has not had an opportunity of determining."—*London Lancet.*

